# Supplementary material for: Construction and validation of a predictive model for the risk of malnutrition in hospitalized patients over 65 years of age with malignant tumours: a single-centre retrospective cross-sectional study
Source: PeerJ. 2024 Dec 10;12:e18685. doi: 10.7717/peerj.18685 (PMC11639871; doi:10.7717/peerj.18685)
Supplement: Supplemental Information 1 [file peerj-12-18685-s001.docx]

Supplementary Table 1: Characteristics of patients aged 65 or older with malignancies

| **Variable** | **Description** | **Number (%)** |
| --- | --- | --- |
| **Total Patients (≥65 years)** | **Total number of patients aged 65 or older with malignancies** | **3,387 (100.0%)** |
| **Malnourished Patients** | **Total number of malnourished patients aged 65 or older** | **1,365 (40.4%)** |
| **Weight Loss** | **Weight loss >5% in the past 6 months** | **541 (39.6%)** |
| **Low BMI** | **BMI <18.5 kg/m²** | **335 (24.5%)** |
| **Decreased Muscle Mass** | **Reduced muscle mass as assessed by skinfold thickness and circumference measurements** | **483 (35.4%)** |
| **Reduced Food Intake** | **Food intake <50% for >1 week or reduced intake for >2 weeks** | **398 (29.2%)** |
| **Inflammation (CRP Elevated)** | **CRP level ≥10 mg/L** | **548 (40.1%)** |
| **Inflammation (ESR Elevated)** | **ESR ≥30 mm/h** | **689 (50.5%)** |
| **NRS-2002 Score** | **Patients with NRS-2002 score ≥3** | **920 (67.4%)** |
| **Missing Muscle Mass Data** | **Percentage of patients with missing muscle mass data** | **672 (19.9%) of total** |
| **Muscle Mass Data with Complete Records** | **Number of patients with complete muscle mass data** | **693 (20.5%)of total** |
