# Supplementary material for: Construction and validation of a predictive model for the risk of malnutrition in hospitalized patients over 65 years of age with malignant tumours: a single-centre retrospective cross-sectional study
Source: PeerJ. 2024 Dec 10;12:e18685. doi: 10.7717/peerj.18685 (PMC11639871; doi:10.7717/peerj.18685)
Supplement: Supplemental Information 2 — *: Optimal cutoff values were established using receiver operating characteristic (ROC) curve analysis to approximate clinical relevance.#: Routine clinical laboratory reference values were adopted.&: Variable assignments were determined through clinical experience and manual segmentation. [file peerj-12-18685-s002.docx]

Supplementary Table 2: Assignment of variables

| **Characteristics** | Assignment of variables | | | | |  |
| --- | --- | --- | --- | --- | --- | --- |
|  | **0** | **1** | **2** | **3** | **4** | **5** |
| **Age^&^(years)** |  | **65-70** | **71-75** | **76-80** | **81-85** | **≥86** |
| **Gender** |  | **Male** | **Female** |  |  |  |
| **Marital state** |  | **Married** | **Divorced/Widowed/Single** | **Other** |  |  |
| **Hukou** |  | **Rural** | **Urban** |  |  |  |
| **System** |  | **Digestive system** | **Others** |  |  |  |
| **Surgical history (last 12 months)** | **No** | **Yes** |  |  |  |  |
| **TNM stage** |  | **I-III** | **IV** |  |  |  |
| **KPS score^*^** |  | **≥90** | **≤80** |  |  |  |
| **Smoking history** | **No** | **Yes** |  |  |  |  |
| **Alcohol consumption history** | **No** | **Yes** |  |  |  |  |
| **Operation history** | **No** | **Yes** |  |  |  |  |
| **Co-infection** | **No** | **Yes** |  |  |  |  |
| **Hypertension** | **No** | **Yes** |  |  |  |  |
| **Type 2 diabetes mellitus** | **No** | **Yes** |  |  |  |  |
| **Hyperlipidaemia** | **No** | **Yes** |  |  |  |  |
| **Coronary heart disease (CHD)** | **No** | **Yes** |  |  |  |  |
| **Ascites or pleural effusion** | **No** | **Yes** |  |  |  |  |
| **RBC^*^ (10^12^/L)** |  | **≥4** | **＜4** |  |  |  |
| **WBC^#^ (10^9^/L)** |  | **<4** | **4-10.0** | **>10** |  |  |
| **PLT ^#^(10^9^/L)** |  | **<100** | **100-300** | **>300** |  |  |
| **HGB (g/L)^*^** |  | **＞120** | **≤120** |  |  |  |
| **Total bilirubin (µmol/L)^*^** |  | **≤26.0** | **>26** |  |  |  |
| **ALT (U/L)^*^** |  | **≤40** | **>40** |  |  |  |
| **AST (U/L)^*^** |  | **≤35** | **>35** |  |  |  |
| **Creatinine (µmol/L)^*^** |  | **≤81** | **>81** |  |  |  |
| **Urea (mmol/L)^*^** |  | **≤8.8** | **>8.8** |  |  |  |
| **NLR^*^** |  | **≤3.24** | **>3.24** |  |  |  |

*: Optimal cutoff values were established using receiver operating characteristic (ROC) curve analysis to approximate clinical relevance.

#: Routine clinical laboratory reference values were adopted.

&: Variable assignments were determined through clinical experience and manual segmentation.
