# Supplementary material for: Construction and validation of a predictive model for the risk of malnutrition in hospitalized patients over 65 years of age with malignant tumours: a single-centre retrospective cross-sectional study
Source: PeerJ. 2024 Dec 10;12:e18685. doi: 10.7717/peerj.18685 (PMC11639871; doi:10.7717/peerj.18685)
Supplement: Supplemental Information 3 [file peerj-12-18685-s003.docx]

Supplementary Table 3. Univariate Logistic Regression Analysis Identifying Risk Factors for Malnutrition in the Training Cohort

| **Characteristics** | **OR (95%CI)** | ***P*** |
| --- | --- | --- |
| **Demographic characteristics** |  | |
| **Age (years)** |  | |
| **65-70** | **Reference** | |
| **71-75** | **1.167(0.958-1.421)** | **<0.001** |
| **76-80** | **1.362(1.088-1.706)** | **0.126** |
| **81-85** | **2.966 (2.297-3.830)** | **0.007** |
| **≥86** | **5.522(3.608-8.451)** | **<0.001** |
| **Marital state** |  | |
| **Married** | **Reference** | |
| **Other (Divorced/Widowed/Single)** | **1.355(1.115-1.647)** | **0.002** |
| **Hukou** |  | |
| **Rural** | **Reference** | |
| **Urban** | **0.562(0.478,0.660)** | **<0.001** |
| **System** |  | |
| **Others** | **Reference** | |
| **Digestive system** | **1.295(1.107-1.515)** | **0.001** |
| **TNM staging** |  | |
| **Stage I–III** | **Reference** | |
| **Stage IV** | **1.110(1.068-1.154)** | **<0.001** |
| **Surgical history (last 12 months)** |  | |
| **No** | **Reference** | |
| **Yes** | **0.723(0.599-0.872)** | **0.001** |
| **KPS score** |  | |
| **≥90points** | **Reference** | |
| **≤80 points** | **5.056(4.248-6.018)** | **<0.001** |
| **Co-infection** |  | |
| **No** | **Reference** | |
| **Yes** | **1.610(1.320-1.911)** | **<0.001** |
| **Hypertension** |  | |
| **No** | **Reference** | |
| **Yes** | **0.519(0.442-0.610)** | **<0.001** |
| **Type 2 diabetes mellitus** |  | |
| **No** | **Reference** | |
| **Yes** | **0.758(0.616-0.932)** | **0.009** |
| **Coronary heart disease (CHD)** |  | |
| **No** | **Reference** | |
| **Yes** | **0.777(0.614-0.983)** | **0.036** |
| **Ascites or pleural effusion** |  | |
| **No** | **Reference** | |
| **Yes** | **2.273(1.766-2.927)** | **<0.001** |
| **Clinicopathological characteristics** | | |
| **WBC (109/L)** |  | |
| **<4** | **Reference** | |
| **4–10** | **1.056(0.861-1.295)** | **0.603** |
| **>10** | **1.796(1.362-2.368)** | **<0.001** |
| **RBC (10^9^/L)** |  | |
| **<4** | **Reference** | |
| **≥4** | **0.475(0.405-0.557)** | **<0.001** |
| **PLT (10^9^/L)** |  | |
| **<100** | **Reference** | |
| **100–300** | **0.777(0.579-1.041)** | **0.091** |
| **>300** | **1.374(0.957-1.972)** | **0.085** |
| **HGB (g/L)** |  | |
| **≤120** | **Reference** | |
| **>120** | **0.349(0.296-0.412)** | **<0.001** |
| **Total bilirubin (µmol/L)** |  | |
| **≤26** | **Reference** | |
| **>26** | **1.599(1.201-2.128)** | **<0.001** |
| **AST (U/L)** |  | |
| **≤35** | **Reference** | |
| **>35** | **1.487(1.227-1.801)** | **<0.001** |
| **Creatinine (Cr; µmol/L)** |  | |
| **≤81** | **Reference** | |
| **>81** | **1.255(1.066-1.476)** | **0.006** |
| **Urea (mmol/L)** |  | |
| **≤8.8** | **Reference** | |
| **>8.8** | **1.624(1.302-2.027)** | **<0.001** |
| **NLR** |  | |
| **≤3.24** | **Reference** | |
| **>3.24** | **2.220(1.897-2.598)** | **<0.001** |
